# Evaluating DBP Formation in Chlorinated Drinking Water: Effects of Contact with System Materials

**DOI:** 10.1021/acsestwater.5c01355

**Published:** 2026-02-26

**Authors:** David Langenbach, Cynthia Kalweit, Dominik Kaczmarek, Aki S. Ruhl

**Affiliations:** a German Environment Agency (UBA) Section II 3.3, Schichauweg 58, Berlin 12307, Germany; b Water Treatment, Technische Universität Berlin, KF4, Straße des 17. Juni 135, Berlin 10623, Germany; c German Environment Agency (UBA) Section II 3.4, Heinrich-Heine-Str. 12, Bad Elster 08645, Germany

**Keywords:** drinking water, pipes, plastic, disinfection byproducts, leaching, epoxy resin

## Abstract

Climate-driven challenges probably increase disinfection in drinking water systems. Interactions between disinfectants and infrastructure materials remain understudied. This study quantifies the consumption of chlorine, the release of dissolved organic carbon (DOC), and the formation of regulated disinfection byproducts (DBPs) from 20 common materials: polymeric pipes, seals, fittings, epoxy resins, and cement mortar. The materials were pulverized to maximize the surface area and create a worst-case scenario. The results were compared with standardized migration tests. Chlorine depletion (>90%) was observed in the waters exposed to epoxy resins, seals, and cement. The formation of DBP, especially trichloromethane (TCM), exceeded 100 μg/L in samples of epoxy resins, cement, vulcanized fiber, and polyamide; TCM was detected in polyethylene pipe materials at concentrations between 5 and 18 μg/L. Increased temperatures enhanced DOC leaching and THM formation. Relations between DOC and DBP concentrations indicate material leachates as precursors. The results highlight the importance of material selection and testing for disinfected drinking water systems, providing critical insights for risk assessment, material certification, and regulatory development.

## Graphical abstract

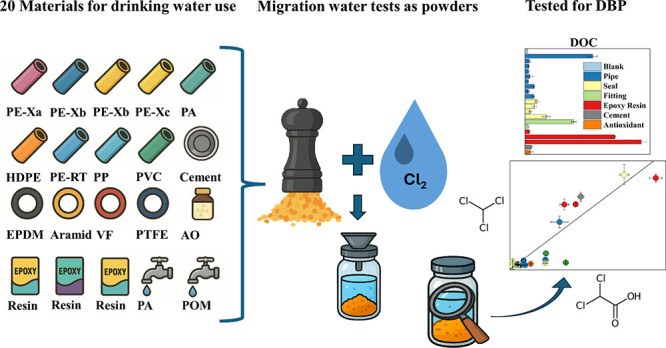

## 1 Introduction

The safety and quality of drinking water during distribution are influenced by its interactions with various infrastructure materials – such as polymeric pipes, fittings, seals, valves, cementitious linings, or epoxy-based coatings – which might release migrating substances into the water. For this reason, intensive research has focused on the migration of substances from commonly used materials into drinking water for decades. In household installations, pipe materials used include polyvinyl chloride (PVC), polybutene (PB), high density polyethylene (HDPE), and polypropylene (PP).^1–4^ Among these, polyethylene (PE) – especially cross-linked PE (PE-X) – is increasingly preferred due to its flexibility, durability, thermal stability, and corrosion resistance. PE-X materials are classified according to their cross-linking methods: PE-Xa with peroxide cross-linking, PE-Xb with silane grafting, and PE-Xc with irradiation.^5–7^ To enhance stability during manufacturing and final use, antioxidants and other stabilizers are typically added.^8^ However, the risk of diffusion of unexpected organic substances into the drinking water – as a result of chain scission and antioxidant degradation during the manufacturing process – is high and may impair drinking water quality.^9–13^ Compounds such as low molecular mass ketones, aldehydes, alcohols, and carboxylic acids,^14,15^ together with residual monomers and oligomers have been reported in the literature as possible oxidative degradation products.^7,13,16^

While research has predominantly focused on pipe materials, much less is known about fittings and seals, often made from organic materials such as polyoxymethylene (POM), polyamide (PA), ethylene-propylene-diene monomer rubber (EPDM), and polytetrafluoroethylene (PTFE).^4,17,18^ Despite their relatively small surface areas, these components may still release degradation products into water. Another relevant class of materials is epoxy-based coatings used in pipe relining: These can leach endocrine-disrupting compounds such as bisphenol A (BPA), raising concerns about long-term exposure.^19–21^

The growing challenge of climate change (e.g., elevated temperatures and thus biological activity in pipes) is prompting water utilities to reconsider distribution strategies, including the adoption of disinfection practices. Disinfection, particularly with chlorine-based agents, is prevalent, economical, and effective for microbial control but can lead to the formation of disinfection byproducts (DBP), some of which are regulated because of toxicological concerns.^22–26^ While DBP formation with natural organic matter (NOM) has been extensively studied, relatively few investigations have examined how disinfectants interact with distribution system materials. Chlorine may react with organic matter leached from materials or directly degrade the materials through oxidation, potentially affecting both infrastructure integrity and drinking water quality.^27^

Previous studies have demonstrated material-specific DBP formation under disinfection. Cao et al. demonstrated that trihalomethane (THM) formation from PE-X pipes was relatively low at 22 °C but doubled by an increase to 55 °C in PE-Xa systems, highlighting the influence of temperature and material type.^28^ Similarly, Elsharkawy et al. reported significant chlorine consumption and DBP formation from an EPDM rubber seal leachate in drinking water systems. These findings underscore the need to expand beyond pipes to other components.^29^

While Southern European countries have long employed disinfection practices, such approaches are less common in countries like Germany. Moreover, to our knowledge, no systematic studies have assessed the impact of disinfection on materials beyond piping, such as fittings, seals, valves, cementitious linings, or epoxy-based coatings. This lack of comprehensive risk assessment hampers the development of effective disinfection strategies, the selection of compatible materials, and the support of evidence-based policymaking. Therefore, there is an urgent need for targeted research to evaluate the interactions between disinfectants and the full range of materials used in drinking water distribution systems, especially with regard to DBP formation and material degradation.

This gap was addressed by evaluating the effects of disinfection on the worst-case scenario of DBP formation potential from commonly used materials in the drinking water distribution system. Twenty materials commonly found in European drinking water systems were selected for this study. These materials were newly purchased and unused and were chosen to represent those with varying surface areas that come into contact with drinking water. The materials included ten plastic pipes, a commonly used antioxidant (Irganox 1010), three seals, two fittings, three epoxy resins, and a pipe lined with cement mortar. These materials were investigated in suspension for their chlorine consumption, organic matter release under chlorinated and nonchlorinated conditions, and DBP formation potential. In the present study, all materials were pulverized for an alternative test procedure besides the standard method^30^ to maximize the surface area and to enhance compound releases, thus simulating a worst-case exposure scenario.

## 2 Materials and Methods

### 2.1 Chemicals and Materials

Sodium hypochlorite solution (NaOCl) with 6–14% active chorine was purchased from Merck (Darmstadt, Germany). All stock solutions were prepared daily and stored in headspace-free amber glass vials at 4 °C. Ultrapure water was obtained from a Merck Milli-Q IQ 7000 Ultrapure Water Purification System (Darmstadt, Germany). Irganox 1010 (Sigma-Aldrich, USA) (3,5-di-*tert*-butyl-4-hydroxyhydrocinnamate) (98%) is a commonly used antioxidant in the production of polymer pipes.^31,32^ Ethylenediaminetetraacetic acid disodium salt dihydrate (99%), potassium iodate (99.8%), disodium hydrogen phosphate (98%), and potassium phosphate (99%) were obtained from Merck. *N*,*N*-Diethyl-1,4-phenylendiaminsulfate (DPD) (98%) was obtained from Sigma-Aldrich.

A total of 20 materials were examined that can be classified into six categories: pipes, fittings, seals, epoxy resins, cement, and antioxidant (see Table 1). All were accredited by the national certification bodies for drinking water use. The pipes, seals, and fittings were purchased from local German providers and stored at room temperature in a dark place. The pipes were sealed with caps or aluminum foil.

**Table 1:** Types (Six Categories) and Abbreviations of All Materials Tested in the Migration Water Tests with Surface to Volume (*S*/*V*) Ratios for Pipes

type	abbreviation	material	*S*/*V* (dm^–1^)
pipe	PE-Xa	peroxide-PE-X	34
pipe	PE-Xb_s_	silane-PE-X^a^	40
	PE-Xb_b_		25
pipe	PE-Xc	irradiation-PE-X	31
pipe	PA	polyamide	34
pipe	PE-RT	PE of raised temperature resistance	31
pipe	HDPE	high-density PE	34
pipe	PP	polypropylene	30
pipe	PVC	polyvinyl chloride	33
fitting	PA	polyamide	
fitting	POM	polyoxymethylene	
seal	EPDM	ethylene-propylene-diene rubber^b^	
seal	Aramid	Aramid	
seal	VF	vulcanized fiber	
seal	PTFE	polytetrafluoroethylene	
coating	ER 1	epoxy resin	
coating	ER 2	epoxy resin	
coating	ER 3	epoxy resin	
coating	cement	cement mortar (CEMIII/A)^c^	
additive (antioxidant)	Irganox 1010	pentaerythritol-tetrakis(3,5-di-*tert*-butyl-4-hydroxyhydrocinnamate)	

a Two PE-Xb pipes with a smaller inner diameter of 10 mm and with a bigger inner diameter of 16 mm were tested.

b Peroxide cross-linked EPDM.

c Blast furnace cement according to standard.^33^

### 2.2 Pulverizing Materials

All materials, including pipes, were ground into powder, except for Irganox 1010, which was supplied in powder form. The materials were pretreated according to the respective standards^30^ and dried before milling. The internal components of the pipes and other materials, i.e., the layer that comes into contact with drinking water during use, were meticulously divided into fragments measuring 3–5 mm. A cryogenic ball mill (Retsch CryoMill, Germany) with a single stainless-steel ball (25 mm diameter) was then used to pulverize 2.2 g of the plastic pieces. The volume of the jar was thus filled to about 1/3 each with the grinding ball, pieces of material, and air. The grinding jar (50 mL) was continually cooled with liquid nitrogen before and during milling. The cooling process was carried out in a sequence of three cycles. The first cycle consisted of (i) a precooling step with a frequency of agitation of the grinding jar of 5 Hz and a duration of 20 min, (ii) a grinding step (frequency 30 Hz, duration 5 min), and (iii) an intermediate cooling step (frequency 5 Hz, duration 5 min). Cycles ii and iii were repeated three times. Further details are reported by Eitzen et al.^34^ After milling, the powders were collected in amber glass vials with a PTFE cap to minimize the loss of volatile organic compounds (VOC) and prevent cross-contamination. The vials were then stored in the dark at room temperature. The mill was also used to grind the cement sample without liquid nitrogen.

The numbers of particles of the resulting plastic powders were determined in a suspension by using a particle counter (PAMAS SVSS, Rutesheim, Germany) based on laser light extinction measurements. The stock suspensions of 500 mL with a concentration of 10 mg/L of the powders were prepared using ultrapure water (ELGA, Celle, Germany), and a nonionic surfactant (NovaChem, Postnova) was used to stabilize the suspension.

### 2.3 Experimental Setup

The glass bottles used for the tests were all washed at least three times with ultrapure water and dried (100 °C for at least 2 h). Glass bottles containing chlorinated samples were soaked in a NaOCl solution (ca. 100 mg/L) for at least 4 h beforehand.

#### 2.3.1 Migration Tests with Powdered Materials

All samples were prepared with ultrapure water (18.2 MΩ) and tested in batch with NaOCl (1 and 10 mg Cl_2_/L) and without disinfection. All migration samples were agitated in glass (10 and 50 mL) with an overhead rotator (Heidolph REAX2, Schwabach, Germany) at a rotational frequency of about 60 rpm. After contact times of 0.25, 1, 24, and 72 h, the residual chlorine concentrations (performed immediately), dissolved organic carbon (DOC), and DBP concentrations were measured.

Additionally, the influence of the temperature was analyzed. The standard for testing materials for drinking water use specifies a temperature range for cold water tests of 23 °C ± 2 °C.^30^ Therefore, migration water tests with selected pipe materials and Irganox 1010 were carried out and compared at 21 and 25 °C ± 0.5 °C for 72 h. Samples intended for THM measurements were quenched with sodium thiosulfate (Na_2_S_2_O_3_). All samples containing powders (except samples for THM measurement) were filtered after 72 h through a prewashed membrane with a pore size of 0.45 μm (CHROMAFIL Xtra PTFE, Macherey-Nagel, Germany) and stored in the dark at 4 °C before analyses. Blank and filter blank samples were prepared accordingly to quantify potential contamination. In addition, blank residual chlorine tests to account for disinfectant decay in water without any material were performed adopting the same procedures used for migration tests.

#### 2.3.2 Conventional Migration Tests

Pipes in pieces of 1.5 m length were left in their manufactured form, sealed with washed and heated-treated glass stoppers, and filled with chlorinated and unchlorinated ultrapure water. The pretreatment steps of rinsing, stagnation, and rerinsing, as well as the first migration period after 72 h, were carried out in accordance with the standard method^30^ for seven of the plastic pipes­(PE-Xa, PE-Xb_s_, PE-Xc, PP, PVC, HDPE, and PE-RT). The water was completely replaced between the stagnation and migration phases. The water from the 72 h migration was taken for analysis at a test temperature of 21 °C.

### 2.4 Analyses

#### 2.4.1 Standard Parameters

The pH was measured by a pH meter (pH 340i, WTW, Weilheim, Germany). Temperatures were measured during the whole migration tests (Testo datalogger 175T1, Lenzkirch, Germany). Absorbance measurements were done with a UV/vis spectrophotometer (Lambda 25, PerkinElmer, USA) using a quartz cuvette with a 10 mm optical path. All measurements of DOC were carried out according to the respective standard with a TOC analyzer (Elementar vario TOC cube, Langenselbold, Germany).^35^ Cl_2_-equivalent concentrations in the NaOCl stock solution were determined according to the iodometric method.^36^ Residual chlorine in samples was measured using the standardized DPD colorimetric method.^37^ Additionally, blank decay tests were performed to measure the chlorine decay in water without materials using the same procedures as for migration tests.

#### 2.4.2 Trihalomethanes

For all experiments, the concentrations of four THM, namely, trichloromethane (TCM), bromodichloromethane (BDCM), chlorodibromomethane (DBCM), and tribromomethane (TBM), were measured with a headspace gas chromatograph coupled to mass spectrometry (HS-GC-MS, Agilent, Santa Clara, USA). The GC (Agilent 7890A) was equipped with a 60 m column (HP-5 ms Ultra Inert, 19091S-436UI, Agilent) with an inner diameter of 0.25 mm and a 0.25 μm film thickness. The MS (Agilent 5975C) was operated in single ion monitoring mode and used an electron beam for ionization while the ions were filtered with a quadrupole. The headspace sampling was performed by a system (Gerstel MPS) using 20 mL amber glass bottles. Samples were shaken for 40 min at 60 °C for equilibration. For injection, 1 mL of gas sample was taken with a split of 1:10 at 210 °C in a split/split-less injector. The samples were analyzed according to the temperature program described in Table SI1 and detected by SIM (82, 83, 84, 85, 93, 117, 119, 124, 127, 129, 161, 163, 171, 173, and 174 *m*/*z*). The limit of quantification was 1 μg/L based on a 3:1 signal-to-noise ratio for this concentration.

#### 2.4.3 Haloacetic Acids

For this study, eight haloacetic acids (HAA8) including trichloroacetic acid (TCAA), bromodichloroacetic acid (BDCAA), chlorodibromoacetic acid (CDBAA), dichloroacetic acid (DCAA), bromochloroacetic acid (BCAA), dibromoacetic acid (DBAA), monochloroacetic acid (MCAA), and monobromoacetic acid (MBAA) were quantified by high-performance liquid chromatography (Agilent 1290 Infinity II) coupled with triple quadrupole mass spectrometry (HPLC-MS/MS). A column (Luna Omega Polar) with 100 mm length, 4.6 mm inner diameter, 3 μm particle size, and 100 Å pore size was used. The chromatograph was operated at a gradient program described in the SI, with ultrapure water with 0.1% acetic acid (solvent A) and pure methanol (solvent B) as the eluent, while maintaining a column temperature of 40 °C. The connected mass spectrometer (QTRAP 6500+, Sciex) used electron spray ionization (ESI) in negative mode (CUR 35, CAD High, IS −4500, TEM 600, GS1 50, GS2 50, EP −10). The limit of quantification was 0.1 μg/L for DCAA and 0.5 μg/L for the other seven HAA based on the minimum reporting levels for a signal-to-noise ratio of 3:1.

## 3 Results and Discussion

### 3.1 Chlorine Consumption

Material samples in powdered form were tested for their chlorine consumption after 0.25, 1, 24, and 72 h. The kinetics of chlorine consumption varied significantly among the tested materials (Figure 1). The fastest kinetics occurred in PA (fitting), Aramid, and EPDM materials, which exhibited negligible residual chlorine concentrations after 15 min, which indicates a rapid initial reaction between the material and the chlorine. For the resin materials, cement, and VF, complete chlorine consumption was observed, with a rapid depletion in the first 15 min followed by slower kinetics up to 72 h. In contrast, the plastic pipe materials underwent slower chlorine consumption kinetics. Although the reaction was initially characterized by a rapid decay, the residual concentration after 72 h was more than 50% of the initial concentration of 1 mg/L Cl_2_, with the exception of PA (0.1 mg/L Cl_2_ after 72 h) and PE-Xb_b_ (0.31 mg/L Cl_2_). Residual chlorine in the control blanks remained higher (0.73 mg/L Cl_2_ after 72 h) than in any powdered material samples. Therefore, the observed disinfectant consumption may be related with the release of organic matter from the tested materials into the water (including degradation products) and/or with the reaction of the disinfectant directly with the solid phase of the particles.^20,27,38^ Since the materials were tested in powdered form and remained in suspension by shaking during exposure, the measured chlorine decay most likely reflects predominantly initial surface leaching together with concurrent reactions at particle surfaces, while longer-term diffusion-controlled release from the material matrix would require extended contact times. A study by Liu et al. suggests that leached substances contribute more to chlorine consumption than chlorine reacting with the particle surfaces.^39^

**Figure 1:**
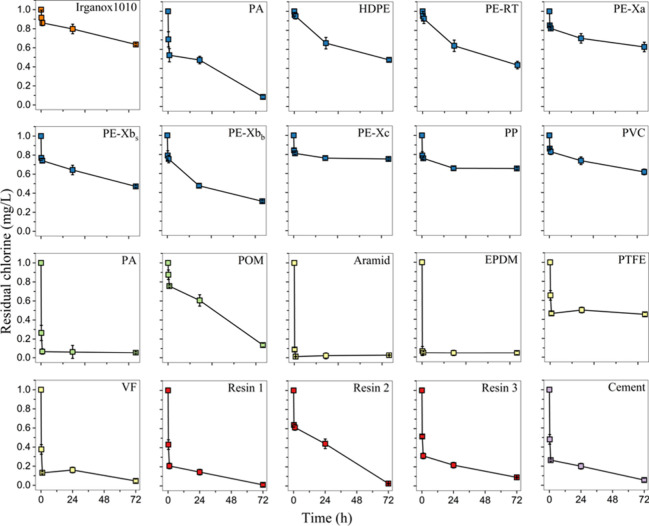
Chlorine decay determined in migration test water of powdered materials at 21 °C.

### 3.2 Leaching of Organics

The observed chlorine consumption is most likely due to organic matter being leached from the materials being tested into the water.^39^ Both chlorinated and nonchlorinated filtrated batches showed a DOC increase for all materials after 72 h contact time compared to blank samples as shown in Figure 2. The DOC concentration indicates that organic matter leached from the materials into the water. With the exception of PA, the chlorinated samples exhibited a minimal higher DOC concentration (on average 15%) than the one measured in the nonchlorinated samples. This can be explained by oxidative cleavage of polymer (and side) chains on the surface resulting in low-molecular, water-soluble oxidation products.^40,41^ Additionally, the increased DOC content may be due to the degradation or conversion of additives, forming more soluble, chlorinated transformation products.^13,42^ Adding NaOCl can also cause oxidation, roughening the surface and forming pores or releasing small particles.^43,44^ This increases the effective surface areas and migration rates of other organic substances. In line with the findings of Chen et al, the epoxy resins 2 and 3 (13.3 and 17.2 mg/L) and PA fitting (7.2 mg/L) samples resulted in the highest DOC concentrations in nonchlorinated water.^45^ Samples containing plastic pipes showed significantly lower DOC values than the other material groups. Lin and Su demonstrated the stimulating effect of chlorination on the leaching of organic substances from PE microplastics.^46^ Liu et al. tested different microplastics (MP) where PE showed the highest resistance to fragmentation while the fragmentation during chlorination increased with reaction time and could contribute to a higher DOC in chlorinated samples.^39^

**Figure 2:**
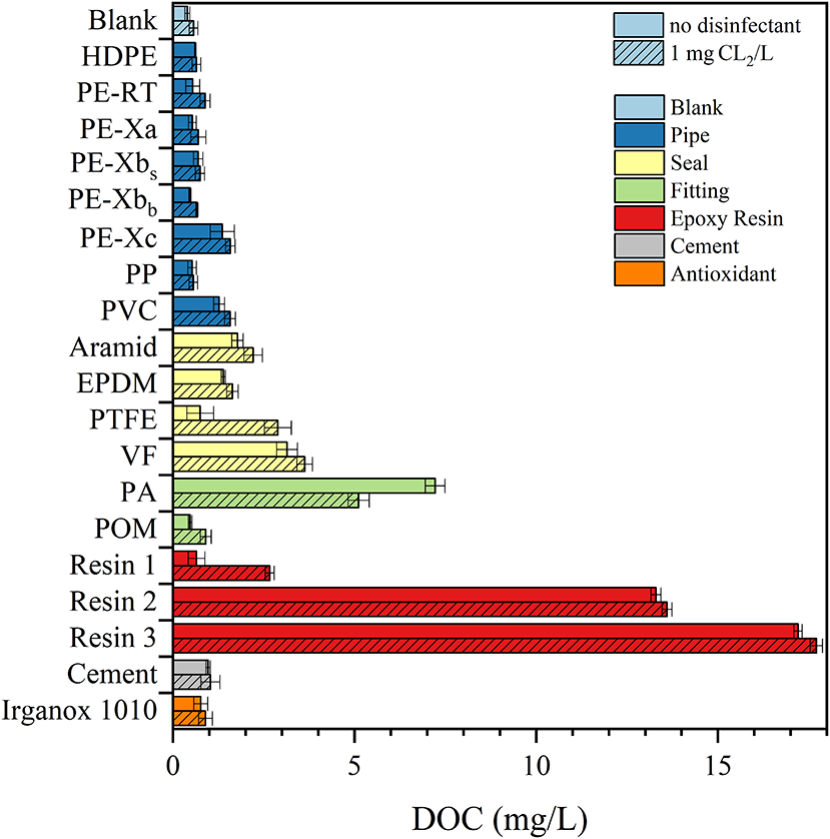
DOC concentrations in filtered migration water samples of the powdered materials after a contact time of 72 h without NaOCl (solid) and with 1 mg/L Cl_2_ (hatched).

#### 3.2.1 Influence of Temperature

Migration water tests conducted with powdered organic pipe materials (PE-Xa, PE-Xb, PE-Xc, PP, PA, HDPE), an EPDM seal, and Irganox 1010 were carried out at 21 and 25 °C ± 0.5 °C. The contact time was 72 h. All samples analyzed at elevated temperatures exhibited increased chlorine consumption (on average 22%) as shown in Figure 3. This finding highlights the critical role of temperature in the final results and indicates the importance of selecting appropriate test temperatures in material testing, given the expected increase in water temperatures due to climate change. It also demonstrates that the requirement for disinfection will result in greater chlorine usage in drinking water distribution systems. Of all of the materials investigated, only EPDM showed an almost complete chlorine consumption at both temperatures. The impact of temperature on chlorine consumption was highlighted in a model presented by García-Ávila et al., who showed that higher monthly average temperatures were associated with faster residual chlorine decay.^47^

**Figure 3:**
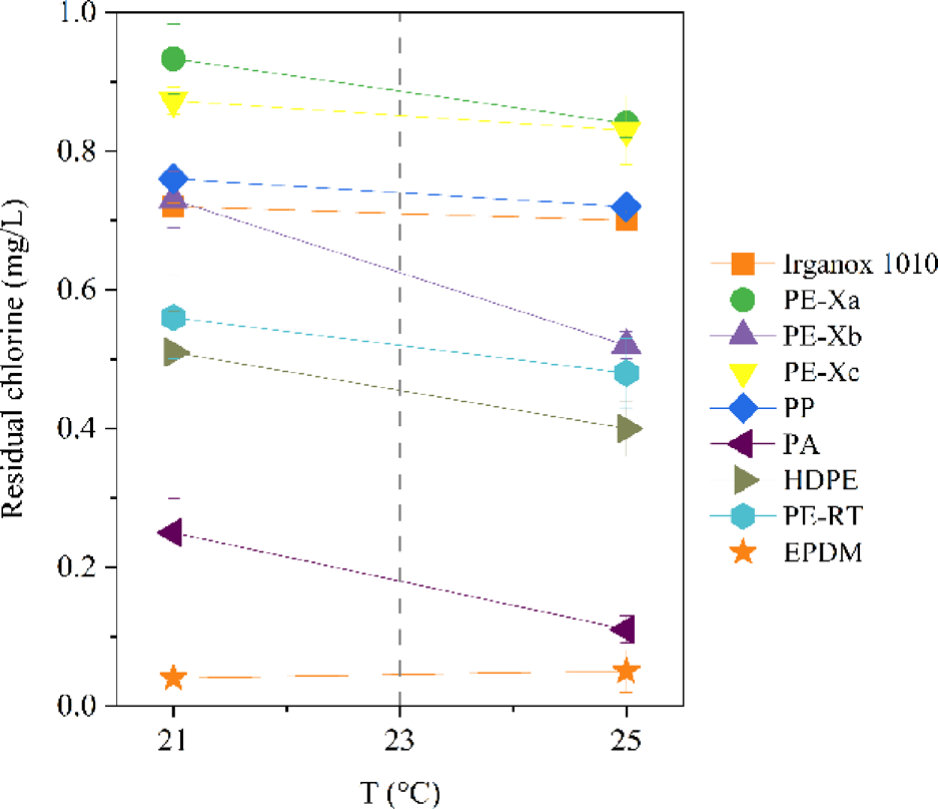
Residual chlorine concentrations after 72 h contact time in batch tests with selected powdered pipe and seal materials and the antioxidant Irganox 1010 at 21 and 25 °C.

Tests were carried out on selected pipe materials (PE-Xa, PE-Xb, PE-Xc, PE-RT, PP, PVC, and HDPE) at 21 and 25 °C ± 0.5 °C to examine the DOC release. The results are shown in Figure 4. The contact time was 72 h. All materials except PVC exhibited increased leaching of organic substances at higher temperatures, a phenomenon also documented by Mathews et al. and Cao et al. for different PE-Xa pipes.^28,48^ The PE-Xb and PE-RT samples showed the biggest increase in DOC at elevated temperatures at +290%. Increased water temperatures (e.g., due to climatic changes) might thus lead to increased leaching of organic matter from pipes in drinking water systems.

**Figure 4:**
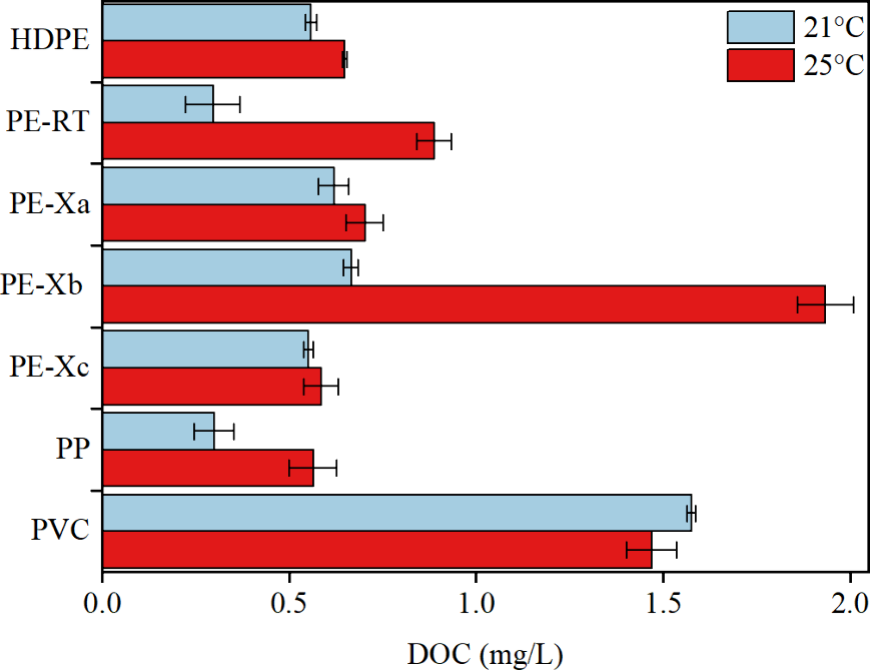
DOC concentrations in migration waters from different pipe materials in powdered form were measured after a contact time of 72 h at 21 and 25 °C without NaOCl.

#### 3.2.2 Comparison of Test Methods

Table 2 shows a comparison of DOC concentrations determined in the migration tests of (1) powdered pipes and the migration tests (2) in accordance to the respective standard (EN 12873–1, 2014).^30^ For all pipes, an increased DOC was measured for powdered materials, which was on average 32% higher than the value obtained using the standard method, indicating that pulverizing material from the inside of the pipe resulted in a higher mass transfer from the solid phase to the aqueous phase.

**Table 2:** Overview of All Materials as Powders and Pipes in the Migration Water Tests at 21°C

	DOC concentrations		TCM concentrations	
	(mg/L)		(μg/L)	
material	powder	pipe	powder	pipe
PE-Xa	0.7 ± 0.1	0.6 ± 0.1	7.7 ± 1.8	3.5 ± 0.5
PE-Xb_s_	0.7 ± 0.0	0.7 ± 0.1	18.0 ± 3.6	16.7 ± 1.7
PE-Xc	0.6 ± 0.0	0.3 ± 0.1	5.6 ± 1.1	5.8 ± 1.1
PP	0.6 ± 0.1	0.3 ± 0.1	6.2 ± 1.6	2.2 ± 0.8
PVC	1.6 ± 0.0	1.5 ± 0.1	16.0 ± 3.7	12.9 ± 0.5
HDPE	0.7 ± 0.0	0.6 ± 0.0	4.5 ± 0.5	4.2 ± 0.3
PE-RT	0.9 ± 0.1	0.3 ± 0.1	5.4 ± 0.3	4.0 ± 0.8

The leaching of DOC from different materials tested could be influenced by the composition of additives, the polymer type, and surface properties. It was reported that old plastic leached more DOC than newer plastics under the same test conditions.^49^ The release of DOC from cement mortar-lined pipes at a measured concentration of 1.2 mg/L in the 72 h migration test is most likely due to the use of organic additives throughout the production process.^50,51^ These additives can account for up to 0.2% of the cement by mass, as specified in the respective standard.^33^

### 3.3 DBP Formation

Although four THMs and eight HAAs were quantified in this study, mainly TCM and DCAA could be detected. Their levels varied significantly among the tested materials as presented in Figure 5, which shows the concentrations in the migration water samples after 72 h contact time with an initial concentration of 10 mg/L Cl_2_. As previously reported in the literature, TCM is the main THM in samples disinfected with NaOCl^52,53^ and can be attributed to the release of organic substances that promote the formation of TCM as precursors.^54,55^ The concentration of TCM detected in the epoxy resin group was the highest (ER1:148 μg/L, ER2:310 μg/L, ER3:149 μg/L), which is consistent with the high DOC release and chlorine consumption by epoxy resins. The formation of TCM was also high in VF migration waters (218 μg/L), which is 15 times higher than the levels quantified in migration waters from other sealing materials. This may be due to the release of its cellulose-derived organic substance, as there was also an elevated DOC in the migration water, in comparison. In the migration water of the cement mortar, 166 μg/L TCM could be quantified. With an average TCM concentration of 106 μg/L, the migration water of PA had the highest concentration among all of the tested pipes. TCM concentrations in the range of 5 to 18 μg/L were quantified in batches of the other pipe materials. Kelley et al. reported substantial DBP formation potential from organic leaching by PE-X pipes in chlorinated water, with major brand-specific differences across PE-Xa, PE-Xb, and PE-Xc types.^56^ In addition to TCM, BDCM was found in samples of VF (0.5 μg/L) and cement (1.8 μg/L) and DCAN in samples of VF and Aramid.

**Figure 5:**
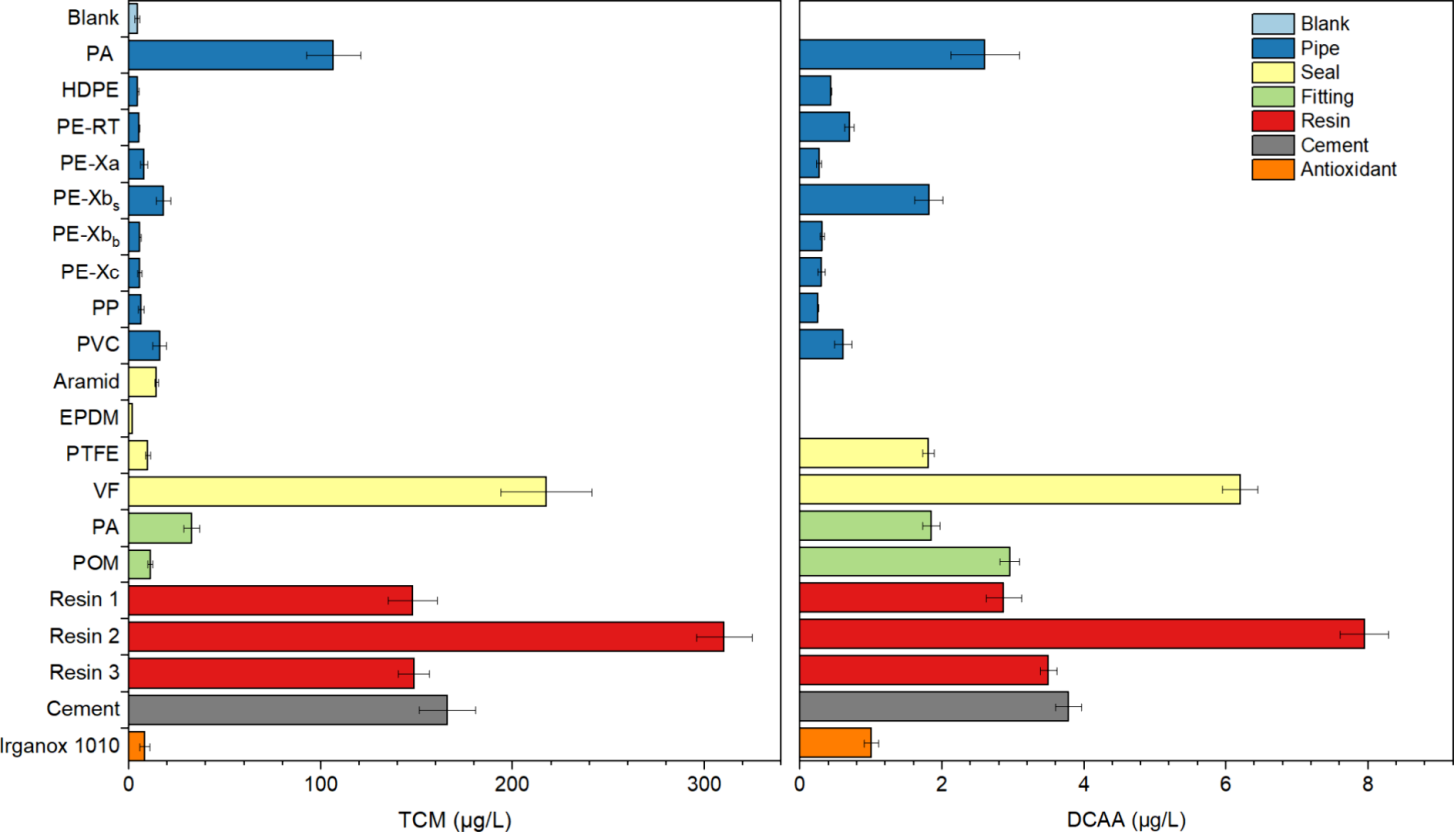
Concentrations of trichloromethane (TCM) (left) and dichloroacetic acid (DCAA) (right) in migration waters of powdered materials after 72 h of contact time with 10 mg/L Cl_2_ at 21 °C.

Analogous to the THM measurements, only one species of HAA (DCAA) was detected in almost all of the samples. The concentrations were significantly lower than those of THM and ranged between 0.25 and 7.95 μg/L. Similar to the TCM amounts in the samples, the three epoxy resins also showed high DCAA concentrations, with the highest concentration in the sample of resin 2. In the samples of the pipes, PA (2.6 μg/L) and PE-Xb (1.8 μg/L) had the highest concentrations of DCAA. Among fitting materials, POM (3.5 μg/L) showed the highest level. Additionally, to DCAA, also BAA was measured with 0.6 μg/L in samples with powder of cement mortar. Regulated DBPs such as THMs and HAAs, which were investigated in this study, may only account for a small proportion of the total DBP concentration and cytotoxicity in chlorinated water.^57,58^ Elastomer materials in drinking water can be sources of unregulated N-nitrosamines such as NDMA, as Morran et al. have demonstrated.^59^

The temperature impact on DBP formation was also investigated. With regard to the measurement of DOC and residual chlorine, the TCM content was also measured at 21 and 25 °C during the migration phase for the seven plastic pipes as powders. After 72 h, the mean values measured at 25 °C were found to be 9% higher than those measured at 21 °C. In the samples PE-Xb_s_ (19.3 μg/L), PP (7.1 μg/L), and PVC (18.1 μg/L), the highest TCM values were measured at an elevated temperature. In almost all samples, increasing water temperature significantly enhanced the release of DOC, as showed in Section 3.2.1, and, hence, promoted the formation of THM during disinfection processes, which is consistent with the findings by Cao et al., who observed a double in THM concentrations at 55 °C compared to 22 °C in PEX-a pipes.^28^

THM formation potential in the migration water of the powdered material was compared to the formation potential in migration water of pipes, prepared in accordance to the respective standard (EN 12873–1, 2014). The results are presented alongside the DOC concentrations in Table 2.

The two tests were conducted simultaneously at 21 °C ± 0.5 °C. On average, the THM concentrations of samples from powders were 22% higher than that of pipe samples, indicating that pulverizing the materials resulted in higher DBP formation. This can be explained by the increase of mass transfer from the solid polymer phase to the aqueous phase and reactions with the solid material. The only exceptions were samples from PE-Xc, where the concentrations of TCM were higher for the pipe (5.8 μg/L) than for the powder (5.6 μg/L).

When comparing the THM and HAA concentrations, it is noticeable that the highest values were found for epoxy resins, VF and PA. However, the concentrations of TCM were much higher than those for DCAA, with over 20 times higher values in average. Similar findings were also documented in other studies.^49,60^ Figure 6 shows the relationship between the TCM and DCAA concentrations. Higher concentrations of DCAA were observed alongside higher concentrations of TCM. The plotted line revealed a slope of 32 and an *R* ^2^ value of 0.81, indicating a positive correlation. Although THM and HAA are highly correlated, DOC shows a moderately positive correlation with both THM and HAA, as well as with residual chlorine (see Figure SI7). The highest values in total of THM and DCAA are found in samples of resin 2 and the PA fitting.

**Figure 6:**
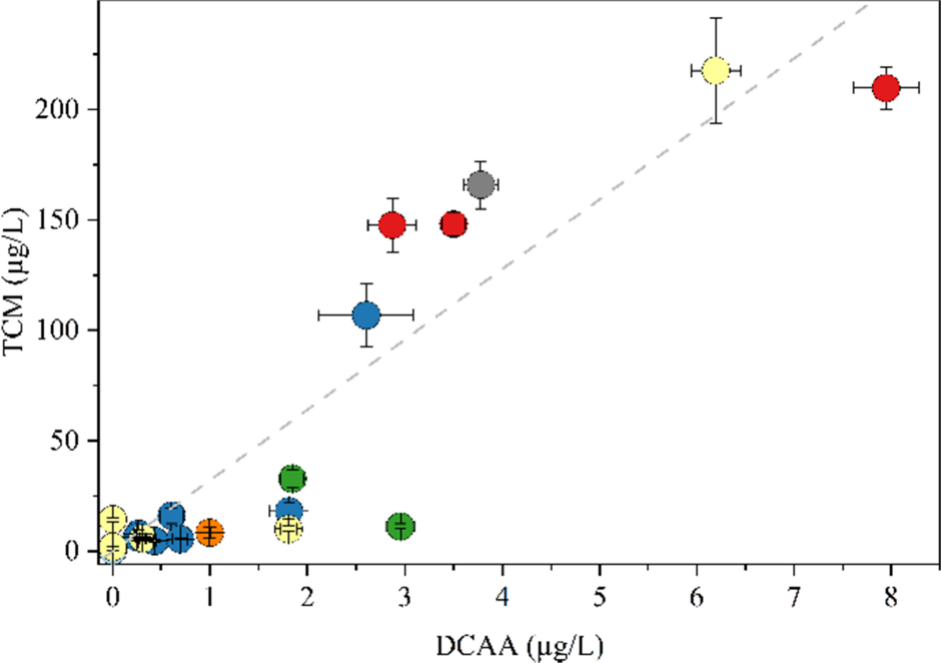
Correlation between DCAA and TCM concentrations determined in migration waters of powdered materials (contact time: 72 h, initial NaOCl: 10 mg/L Cl_2_).

## 4 Conclusions

This study systematically examined the interactions between disinfectants and 20 materials commonly used in drinking water distribution systems, revealing their varying contributions to DBP formation. Batch experiments showed that epoxy resins, followed by seals and cement, exhibited the highest chlorine consumption, DOC release, and formation of THM and HAA, whereas plastic pipes demonstrated a markedly lower impact. The strong correlation between DOC and DBP concentrations highlights the important role of material-derived organic matter as a source of precursors during chlorination. Elevated temperatures were found to intensify chlorine consumption, organic carbon leaching, and DBP formation, thereby highlighting the combined effects of climate change and disinfection on water quality. Comparisons between pulverized and standardized migration tests confirmed that an increased surface area enhances both mass transfer and DBP formation potential, thus creating a worst-case scenario for testing purposes. Given the limited transparency in additive formulations, future research should aim to identify specific leachable compounds responsible for DBP formation and develop predictive models linking material composition, chlorine decay, and DBP yields. In addition, field studies with full-scale systems under operational disinfection are recommended. In the future, the occurrence, concentration, and potential health risk of unregulated substances caused by reactions between materials and disinfectant should be investigated.

## Supplementary Material



## Data Availability

Data will be made available on request.

## Notes

Particle size analysis and *S*/*V* ratios (Figure SI1); GC–MS method for THM, temperature program (Table SI1), retention times (Table SI2); HPLC-MS/MS method for HAA, LC gradient (Table SI3), multiple reaction monitoring program (Table SI4); additional figures (SI2–SI6) for DOC, TCM, and DCAA comparison (PDF)
