# Supplementary material for: Evaluating DBP Formation in Chlorinated Drinking Water: Effects of Contact with System Materials
Source: ACS ES T Water. 2026 Feb 26;6(3):1842–50. doi: 10.1021/acsestwater.5c01355 (PMC12993996; doi:10.1021/acsestwater.5c01355)
Supplement: Supplementary file 1 [file ew5c01355_si_001.pdf]

Supporting Information

Evaluating DBP Formation in chlorinated drinking water: effects of contact with system materials

David Langenbach*(a,b), Cynthia Kalweit (c), Dominik Kaczmarek (a,b), Aki S. Ruhl (a,b)

a German Environment Agency (UBA) Section II 3.3, Schichauweg 58, Berlin 12307, Germany

b Technische Universität Berlin, Water Treatment, KF4, Straße des 17. Juni 135, Berlin 10623, Germany

c German Environment Agency (UBA) Section II 3.4, Heinrich-Heine-Str. 12, Bad Elster 08645, Germany

*Corresponding author: david.langenbach@uba.de

Particle size analysis

The number of particles of the plastic powders was determined using a particle counter (PAMAS SVSS, Rutesheim, Germany) based on laser light extinction measurements. Stock suspensions of 500 mL with a concentration of 10 mg/L of the powder were prepared using ultra-pure water (ELGA, Celle, Germany). The sizes of the particles were divided into 32 classes ranging from 1 µm to 200 µm. The distribution of the particle sizes of all the materials is shown in Figure SI1.

To stabilize particles, a non-ionic surfactant (NovaChem, Postnova) was added to the suspension. Each stock suspension was analysed in five repetitions, according to the procedure suggested by Eitzen et al. ([1,2]). The suspension was constantly stirred during the analysis. Between the batches of different particles, the device was rinsed with 5 runs of ultrapure water.

To compare the migration water samples to the methods to DIN EN 12873-1 (2014) [3] and DIN EN 1420 (2016) [4], a similar surface area to volume ratio (S/V ratio) to the one from pipes was chosen for both tests. This involves the ratio between the surface area of the test piece (S) to come into contact with the test water and the volume of test water (V) per decimeter, i.e. in dm^{-1} (from dm^2/dm^3 or dm^2/L).

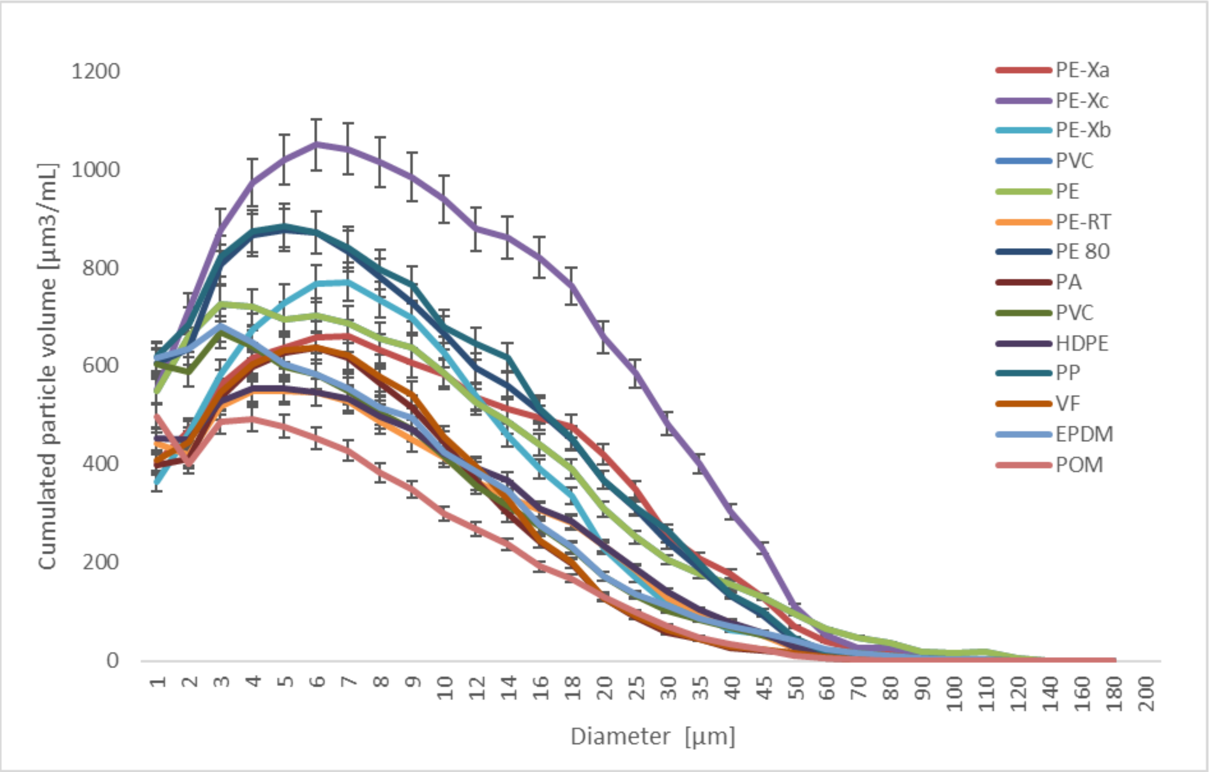

Figure SI1 Particle volume distribution derived from a particle count in the diameter size classes for the cryo-milled plastic samples.

The particle size distributions for the different materials shown in Figure 1 were in similar ranges. The majority of the cumulated volumes of particle diameter classes were in the size range between 5 and 50 µm.

The particle distribution forms the basis for approximating the S/V ratio in the particle suspensions for the migration water tests. An S/V of 33 dm^{-1} was assumed as a comparative value, which corresponds to the average for all pipes not examined. To compare particles with pipes, the simplification was made that all particles have the shape of a sphere. Thus, the specific volume of the powder could be calculated, which resulted in an amount of 700 mg of material per litre of test water at the given S/V ratio.

Trihalomethane

For all experiments the concentrations of trihalomethane (THM) was measured with a headspace gas chromatography and mass spectrometer (HS-GC-MS, Agilent, Santa Clara, USA). The GC (Agilent 7890A) is equipped with a 60 m column (HP-5ms Ultra Inert, 19091S-436UI, Agilent) with an inner diameter of 0.25 mm and 0.25 µm film thickness. The MS (Agilent 5975C) uses an electron beam for ionization while filtering the ions with a quadrupole. The headspace sampling was performed by system (Gerstel MPS) using 20 mL amber glass bottles. The agitator shakes the samples for 40 min at 60°C. 1 mL of gas sample is injected with a split 1:10 at 210°C in a split/split-less injector. The GC is operated with a flow of 0.6 mL/min of helium. The temperature program starts at 40 °C holding for 5 min, rising to 60 °C at 4 °C/min, then to 190 °C at 10 °C/min. The final temperature is hold for another 5 min. The run time per sample was 28 min. The MS operates in single ion monitoring mode scanning for the m/z 82, 83, 84, 85, 93, 117, 119, 124, 127, 129, 161, 163, 171, 173 and 174, each with a dwell time of 80 ms. The retention times are shown in Table SI2. Triplicates were measure according to the respective standard (DIN 38407-30, 2007).

Table SI1. Temperature program of GC for THM measurement, operated with a flow of 0.6 mL/min of helium

	Rate [°C/min]	Temperature [°C]	Hold Time [min]
Initial		40	5
Ramp 1	4	60	0
Ramp 2	10	130	0
Ramp 3	20	190	5

Table SI2. Retention time (RT) of trichloromethane (TCM), bromodichloromethane (BDCM), chlorodibromomethane (DBCM) and tribromomehtane (TBM)

Analyte	RT [min]
TCM	8.18
BDCM	10.92
DBCM	14.03
TBM	15.70

Haloacetic acids

Eight haloacetic acids (HAA8) including trichloroacetic acid (TCAA), bromodichloroacetic acid (BDCAA), chlorodibromoacetic acid (CDBAA), dichloroacetic acid (DCAA), bromochloroacetic acid (BCAA), dibromo acetic acid (DBAA), monochloroacetic acid (MCAA), and monobromoacetic acid (MBAA) were measured by high-performance liquid chromatography (Agilent 1290 Infinity II) coupled with triple quadrupole mass spectrometry (HPLC-MS/MS)

systemA column (Luna Omega Polar) with 100 mm length, 4.6 mm inner diameter, 3 µm particle size and 100 Å pore size was used. The chromatography is operated with ultrapure water with 0.1% acetic acid (solvent A) and pure methanol (solvent B) by a gradient listed in **Error! Reference source not found.**SI3 while maintaining a column temperature of 40 °C. The connected mass spectrometer (QTRAP 6500, Sciex) uses an electron spray ionisation in negative mode (CUR 35, CAD High, IS -4500, TEM 600, GS1 50, GS2 50, EP -10). **Error! Reference source not found.**5 shows the multiple reaction monitoring program (MRM) for all measured quantifiers and qualifiers at the expected retention times. For the calibration, standards with 0.5, 1, 5, 10, 20, 40, 60, 80 and 100 µg/L are measured. The limit of quantification is at 0.5 µg/L for all HAA expect for DCAA at 0.1 µg/L.

Table SI3. Gradient for flushing of LC column for HAA measurements

**Time [min]**	**solvent A [%]**	**solvent B [%]**	**Flow [mL/min]**
0	99	1	0.8
1	99	1	0.8
9	5	95	0.8
10	5	95	0.8
11	99	1	0.8
14	99	1	0.8

Table SI4. MRM program for all measured quantifiers and qualifiers at the expected retention times

	**Quantifier**						**Qualifier**						
**HAA**	**Q1**	**Q3**	**Dwell Time [msec]**	**DP [V]**	**CE [V]**	**CXP [V]**	**Q1**	**Q3**	**Dwell Time [msec]**	**DP [V]**	**CE [V]**	**CXP [V]**	**expected RT [min]**
CAA	93	35	30	-9	-16	-5	95	37	30	-9	-16	-5	2.39
DCAA	127	83	30	-5	-14	-9	129	85	30	-8	-13	-9	2.44
TCAA	161	117	30	-10	-12	-5	163	119	30	-15	-12	-11	4.45
BAA	137	79	30	-10	-22	-7	173	81	30	-15	-18	-9	2.73
DBAA	215	79	30	-10	-36	-9	217	81	30	-20	-34	-9	3.07
TBAA*	251	79	30	-30	-28	-9	253	81	30	-30	-28	-7	5.06
BCAA	171	79	30	-40	-28	-9	173	81	30	-35	-30	-9	3.07
BDCAA*	161	79	30	-5	-18	-7	163	81	30	-15	-18	-9	4.64

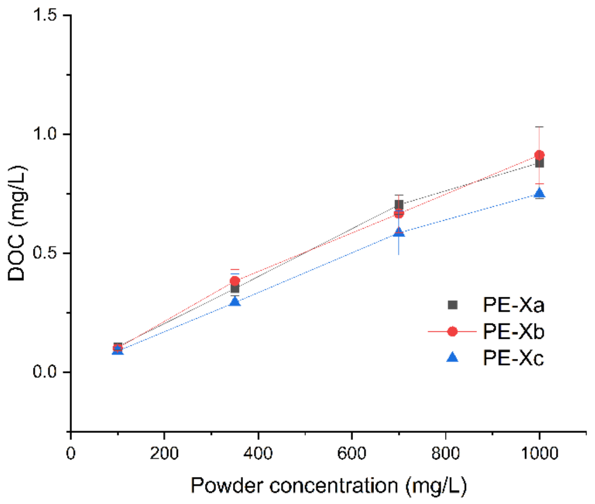

Figure SI2 DOC concentration in the filtered samples of PE-Xa, PE-Xb and PE-Xc in powered form at different concentrations after 72 h at 21 °C.

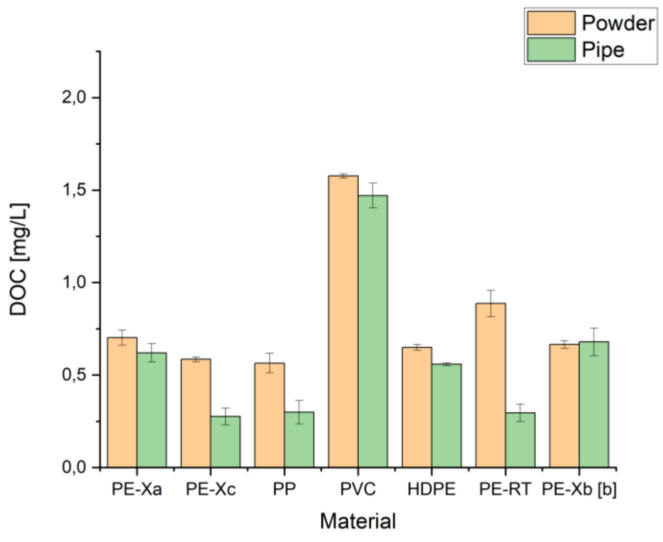

Figure SI3 DOC concentration in the filtered samples of the different materials in powered form and in pipes in comparison

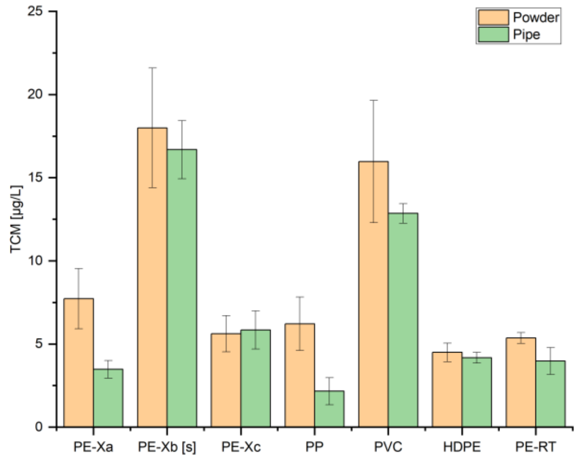

Figure SI4 Amount of TCM in migration water samples by material in comparison of the two different test methods.

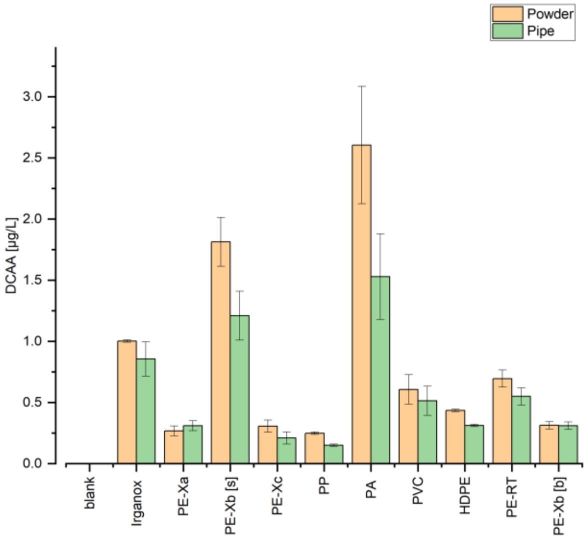

Figure SI5 DCAA concentrations in the migration waters where pipe materials were present in powder form compared to conventional material tests.

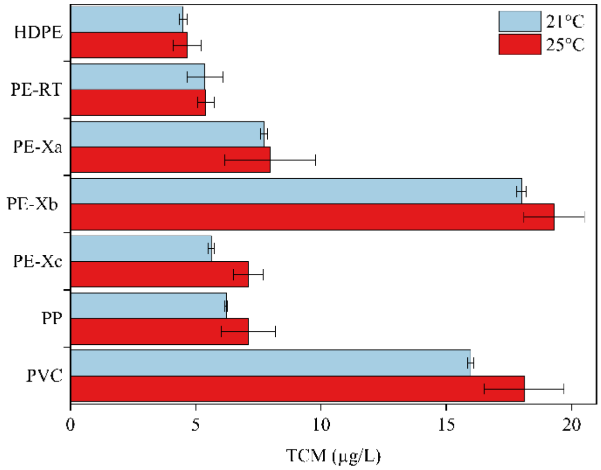

Figure SI6 TCM concentrations in migration waters from different pipe materials in powdered form measured after a contact time of 72 h at 21 and 25°C

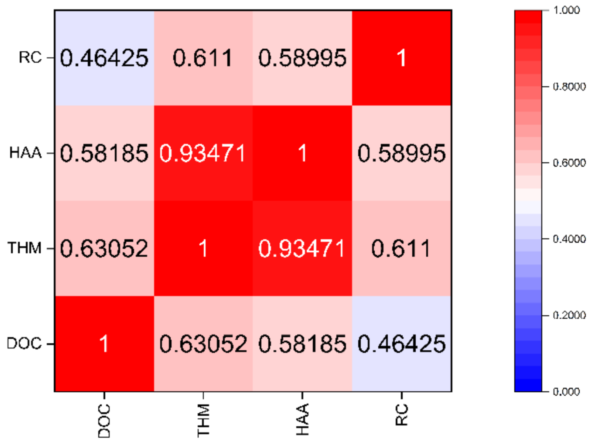

Figure SI7 Pearson correlation matrix between dissolved organic carbon (DOC), total trihalomethanes (THM), total haloacetic acids (HAA) and residual chlorine content (RC72h) after 72 hours of migration (n=20). Significance levels: ***p<0.001, **p<0.01, *p<0.05.

(1) Eitzen, L.; Paul, S.; Braun, U.; Altmann, K.; Jekel, M.; Ruhl, A. S. The Challenge in Preparing Particle Suspensions for Aquatic Microplastic Research. *Environ. Res.* **2019**, *168*, 490–495. https://doi.org/10.1016/j.envres.2018.09.008.
(2) Eitzen, L.; Ruhl, A. S.; Jekel, M. Particle Size and Pre-Treatment Effects on Polystyrene Microplastic Settlement in Water: Implications for Environmental Behavior and Ecotoxicological Tests. *Water* **2020**, *12* (12), 3436. https://doi.org/10.3390/w12123436.
(3) DIN EN 12873-1:2014-09, Einfluss von Materialien Auf Trinkwasser_- Einfluss Infolge Der Migration_- Teil_1: Prüfverfahren Für Fabrikmäßig Hergestellte Produkte Aus Oder Mit Organischen Oder Glasartigen Materialien (Emails/Emaillierungen); Deutsche Fassung EN_12873-1:2014. https://doi.org/10.31030/2088304.
(4) DIN EN 1420:2016-05, Einfluss von Organischen Werkstoffen Auf Wasser Für Den Menschlichen Gebrauch_- Bestimmung Des Geruchs Und Geschmacks Des Wassers in Rohrleitungssystemen; Deutsche Fassung EN_1420:2016. https://doi.org/10.31030/2336872.
(5) DIN 38407-30:2007-12, Deutsche Einheitsverfahren zur Wasser-, Abwasser- und Schlammuntersuchung_- Gemeinsam erfassbare Stoffgruppen (Gruppe_F)_- Teil_30: Bestimmung von Trihalogenmethanen (THM) in Schwimm- und Badebeckenwasser mit Headspace-Gaschromatographie_(F_30), n.d. https://doi.org/10.31030/9878004
